# Breast cancer risk status influences uptake, retention and efficacy of a weight loss programme amongst breast cancer screening attendees: two randomised controlled feasibility trials

**DOI:** 10.1186/s12885-019-6279-8

**Published:** 2019-12-04

**Authors:** Michelle Harvie, Mary Pegington, David French, Grace Cooper, Sarah McDiarmid, Anthony Howell, Louise Donnelly, Helen Ruane, Katharine Sellers, Philip Foden, D. Gareth Evans

**Affiliations:** 1 The Prevent Breast Cancer Research Unit, The Nightingale Centre, Manchester University NHS Foundation Trust, Manchester, M23 9LT England; 2 Manchester Breast Centre, Manchester Cancer Research Centre, University of Manchester, 555 Wilmslow Rd, Manchester, M20 4GJ England; 3 Division of Cancer Sciences, The University of Manchester, Wilmslow Road, Manchester, M20 4BX England; 4 NIHR Manchester Biomedical Research Centre, Manchester Academic Health Science Centre, Central Manchester University Hospitals NHS Foundation Trust, Manchester, England; 5 Manchester Centre for Health Psychology, School of Health Sciences, Manchester Academic Health Science Centre, University of Manchester, Coupland Street, Manchester, M13 9PL England; 6 Department of Medical Oncology, The Christie NHS Foundation Trust, Wilmslow Rd, Manchester, M20 4BX England; 7 Department of Medical Statistics, Education and Research Centre, Manchester University NHS Foundation Trust, Manchester, M23 9LT England; 8 Genomic Medicine, Division of Evolution and Genomic Sciences, The University of Manchester, St Mary’s Hospital, Manchester University NHS Foundation Trust, Oxford Road, Manchester, M13 9WL England

**Keywords:** Breast screening programme, Risk information, Weight loss

## Abstract

**Background:**

Excess body weight and sub-optimal lifestyle are modifiable causes of breast cancer and other diseases. There is little evidence that behaviour change is possible within screening programmes and whether this is influenced by prior knowledge of disease risk. We determined whether breast cancer risk influences uptake, retention and efficacy of a weight control programme in the UK National Health Service Breast Screening Programme, and whether additional cardiovascular disease and type 2 diabetes risk information improves uptake and retention further.

**Method:**

Overweight/obese women in the UK National Health Service Breast Screening Programme identified at high, moderately increased, average and low-risk of breast cancer were randomised to receive individualised breast cancer risk information (breast cancer prevention programme), or individualised breast cancer, cardiovascular disease (QRISK2) and type 2 diabetes (QDiabetes, HbA1c) information (multiple disease prevention programme). Personalised breast cancer risk feedback was given before randomisation in Study-1, and after randomisation in Study-2.

**Results:**

Recruitment was 9% (126/1356) in Study-1 and 7% (52/738) in Study-2. With respect to breast cancer risk, odds ratio of uptake for high/moderately increased vs low risk women was 1.99 (95% CI 1.24–3.17, *P* = 0.004) in Study-1 and 3.58 (95% CI 1.59–8.07, *P* = 0.002) in Study-2. Odds ratio of retention for high/moderately increased -risk vs. low risk women was 2.98 (95% CI 1.05–8.47, *P* = 0.041) in Study-1 and 3.88 (95% CI 1.07–14.04, *P* = 0.039) in Study-2. Weight loss of ≥5% at 12 months was achieved by 63% high/moderate vs. 43% low-risk women in Study-1 (*P* = 0.083) and 39% vs. 8% in Study-2 (*P* = 0.008). Uptake, retention and weight loss were equivalent in both the breast cancer prevention programme and the multiple disease prevention programme in both studies.

**Conclusions:**

Women who are informed that they are at increased breast cancer risk were significantly more likely to join and remain in the programmes and consequently lose more weight across both studies. High risk women are more likely engage in a lifetyle prevention programme and also have the greatest potential benefit fom risk reduction strategies.

**Trial registration:**

ISRCTN91372184 Registered 28 September 2014.

## Background

Maintaining a healthy weight, limiting alcohol, and meeting physical activity (PA) recommendations could prevent 19–26% of breast cancer (BC) in Western populations in the UK [1], Europe [2] and USA [3]. These healthy behaviours could potentially reduce risk of 12 other cancers, type 2 diabetes (T2D) and cardiovascular disease (CVD) [4]. Unhealthy lifestyles are prevalent amongst women in the UK NHS Breast Screening Programme (NHSBSP) [5]. Our recent Predicting Risk of Cancer At Screening (PROCAS) Study reported that 37% of women were overweight and 27% obese, 80% of women had low levels of PA [5] and 11.5% were drinking > 14 units of alcohol/week [5, 6].

The PROCAS Study has been described previously [6]. It assessed the feasibility of collecting breast cancer risk information and providing personalised BC risk assessment amongst 53,000 women in the Manchester NHSBSP between 2009 and 2013. Breast cancer risk assessment was used to triage higher risk patients for chemoprevention, and risk-adapted screening [7].

Previous studies which provided risk of cancer or risk of other diseases either without any lifestyle advice or with simple written lifestyle advice, have not achieved lifestyle behaviour change [8–12]. This is not surprising, since supportive programmes are required to achieve clinically significant behaviour change for individuals [13]. An important but poorly studied research question is whether personalised disease risk information could promote engagement with supportive lifestyle programmes. There is limited information about the acceptability, potential benefits or harms of a multiple disease prevention programme which provides risk information across a number of diseases (breast cancer, CVD and diabetes) [14]. On one hand multiple disease risk information may be more personally relevant to a larger group of women than cancer risk information alone. This may increase the likelihood of the information being responded to, and hence increase engagement overall [15]. Alternatively, multiple disease risk information could reduce motivation and self-efficacy, and threaten self-integrity to change lifestyle behaviours in some individuals, where it could either increase anxiety or “dilute” the message about increased risk of breast cancer linked to weight.

We report two feasibility studies to assess uptake, retention and weight loss in a supported weight loss programme in the context of the NHSBSP and whether the outcomes were related to given (or perceived) BC risk, or altered when women were provided additional information about their personal risk of CVD and T2D.

## Methods

The study adheres to CONSORT guidelines. The study included overweight/obese (BMI ≥25 kg/m [2]) women aged 47–74 years identified at high (10-year risk ≥8%), moderately increased (5–7.9%), average (2–4.9%) or low-risk (< 2%) of BC in the PROCAS study [7] according to NICE criteria [16], as described previously [17]. These women were randomised to receive a mailed invitation to either a BC prevention or multiple disease prevention programme. The BC prevention programme received only information on their personal risk of BC, whilst the multiple disease prevention programme received information on their personal risk of BC, CVD and T2D. BC risk was estimated used the Tyrer-Cuzick model (version 8) which combines family history, hormonal risk factors i.e. age of first pregnancy, menarche and menopause, use of hormone replacement therapy or oral contraceptives, BMI and visually assessed mammographic density [18]. Ten year risk of CVD was assessed using QRISK2 [19] and 10 year risk of T2D with QDiabetes [20], aswell as HbA1c > 42 mmol/mol.

**Fig. 1:**
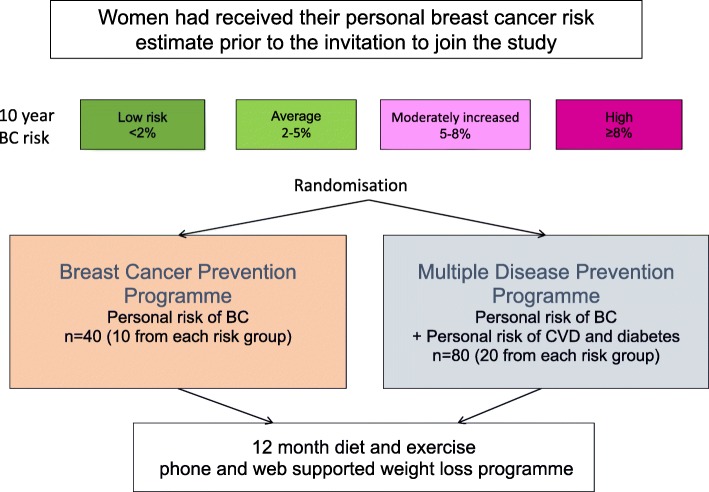
Sampling strategy from PROCAS cohort for Sub study 1. Women were informed of their breast cancer risk before being invited to the study

**Fig. 2:**
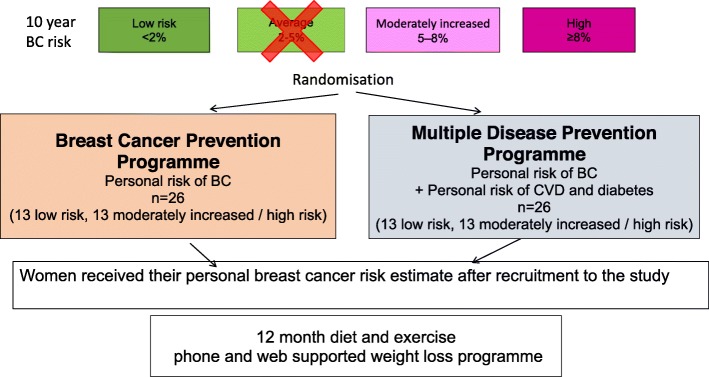
Sampling strategy from PROCAS cohort for Sub study 2. Women were informed of their breast cancer risk after being invited to the study

Key demographic factors which may influence uptake, retention and efficacy were ascertained. The PROCAS cohort had provided information on their ethnic background, and self-reported weight and height, whilst index of deprivation was derived from post codes using Greater Manchester Quintiles [21]. Women who joined the two feasibility studies were categorised as current, ex or never smokers to assess how this influenced retention and efficacy of the programmes. Sampling of PROCAS cohort for the two studies is shown in Fig. 1 (Study 1) and Fig. 2 (Study 2).

Women in Study-1 had already been informed of their BC risk as part of the PROCAS study. This study was designed to examine uptake, retention and weight loss for women who had already been informed they were at high, moderately increased, average or low-risk of BC. Randomisation to invitation to the BC prevention programme or the multiple disease prevention programme was undertaken independently from the research team in the Department of Statistics at Manchester University Hospital Foundation NHS Trust (MFT) using nQuery Advisor 7.0. The first batch of randomisation used a 1:2 randomisation to the BC prevention programme and the multiple disease prevention programme. Adaptive randomisation was used in each subsequent randomisations until we had included 10 women from each BC risk group to the BC prevention programme and 20 from each to the multiple disease prevention programme. The sample size was pragmatic. The 1:2 randomisation allowed us to include greater numbers of women receiving the novel multiple disease risk programme. Thus enabling us to gain information of the feasibility and possible effects of such a programme.

Study-2 invited women who had had not been informed of their BC risk at the time of the invitation, and who received this information during the first 3 months after randomisation. This study was designed to inform uptake in the different BC risk groups according to perceived risk at invitation rather than given risk in Study 1. Also any effects on retention and efficacy once women were informed of their risk. Study 2 invited women who had been identified at high and above average and low risk of breast cancer. We did not include the average risk group, as we wished to assess proof of principle of any effects on uptake and retention to the programme between the highest and lowest risk groups. Women in Study 2 were invited 1:1 to the BC prevention and the multiple disease prevention programmes. This was a pragmatic decision and not based on a sample size calculation. Adaptive randomisation included 13 high/moderately increased risk and 13 low-risk women to both programmes. Women were excluded from both studies if they had a previous diagnosis of cancer, T2D or CVD, or were prescribed statins.

The BC prevention programme included individualised face-to-face diet and PA advice to follow either a daily or intermittent energy restricted diet (2 energy restricted days per week) and meet PA recommendations (> 150 min moderate intensity/week) as described previously [22]. Women received ongoing face to face (week 12 and 26), phone (week 1, 4, 8) and e-mails (week 0–26). Women were advised weight loss of ≥5% and adherence to PA and alcohol recommendations could lead to significant reductions in risk of BC (25%) [23, 24], T2D (60%) [25] and CVD (30%) [26]. The multiple disease prevention programme was identical but also included personalised CVD (QRISK2) and T2D (Qdiabetes and HbA1c) risk information as outlined above.

Trial recruitment and body weight assessments were undertaken at the Prevent BC research centre at MFT using standardised methods as described previously [22]. Recruitment was between November 2014 and October 2016. Twelve month follow up was completed by November 2017. Neither the researchers or the participants were blind to their treatment allocation but researchers were blinded to women’s level of BC risk. This paper reports uptake, retention and weight loss over 12-months to the BC and multiple disease risk programmes and across the four BC risk groups within the two studies.

### Statistical methods

We assessed uptake and retention in both studies in the BC and multiple disease risk groups. Also uptake and retention across the four BC risk groups and in relation to a priori criteria which included index of multiple deprivation (Townsend quintile three groups; 1, 2 and 3–5), ethnic group, smoking status (Chi squared tests). Also mean age and BMI and median (interquartile) for months since women had received BC risk feedback between those who did and did not join or remain in the study (independent sample t-test and Mann Whitney test). Variables with *P* < 0.1 in the single variable analyses were included in a multivariate logistic regression model and presented in the results. Baseline observation carried forward (BOCF) 12 month weight change is reported across the four BC risk groups in terms of kg and the percentage losing ≥5% weight, a level previously associated with reduced BC risk [23]. Statistical significance (2- sided) was accepted at *P* < 0.05. SPSS 22 was used to perform the analysis.

## Results

### Baseline characteristics

Characteristics of women in Study 1 and 2 are reported in Table 1. The cohorts were mainly comparable except women in Study 1 were older than those in Study 2; mean (SD) 59.0 (5.1) vs. 53.3 (4.3) years *P* < 0.005). Women in Study-1 had received their BC risk feedback median (interquartile range) 12.0 (21.6) months previously.

**Table 1:** Baseline demographics

	Study-1 Women informed of their breast cancer risk prior to invite to the weight loss programme	Study-2 Women informed of their breast cancer risk part way through the weight loss programme
Age (years)^a^	59.0 (5.1)	53.3 (4.3)
BMI (kg/m^2^)^a^	31.4 (4.5)	31.1 (4.8)
Townsend quintile (%):		
1 (least deprived)	32	46
2	41	25
3	20	17
4	5.5	8
5 (most deprived)	1.5	4
Current smoker (%)	7	6
Ethnicity (%):		
White British	97	98
Asian	1.5	1
Afro-Caribbean	1.5	1
Number of first degree relatives with breast cancer		
0	68.3	76.9
1	28.6	23.1
> or = 2	3.2	0.0
Time since receiving risk feedback (months)^b^	12.0 (21.6)	N/A

a mean (SD) b median (interquartile range) N/A not applicable as women received their risk feeback after being invited to Study 2

### Breast Cancer and multiple disease prevention Programmes

In Study-1 126/1356 (9%), and in Study-2 52/738 (7%) of women invited agreed to randomisation (Table 2). There was no difference in uptake, retention and weight loss between the breast cancer and multiple disease prevention programmes in either study. (Table 2).

**Table 2:** Uptake, retention and weight loss in the breast cancer and multiple disease prevention programmes in the two studies

	Study-1 Women informed of their breast cancer risk prior to invite to the weight loss programme			Study-2 Women informed of their breast cancer risk part way through the weight loss programme		
	Breast cancer prevention programme	Multiple disease prevention programme	Total/average	Breast cancer prevention programme	Multiple disease prevention programme	Total/average
Invited n	508	848	1356	349	389	738
Uptake n (%)	81 (10%)	45 (9%)	126 (9%)	26 (7%)	26 (7%)	52 (7)%
Retention at 12 months n (%)	33 (73%)	53 (65%)	86 (68%)	12 (46%)	12 (46%)	24 (46%)
Weight loss kg^a^	−6.2 (−7.7 to 4.7) kg	−6.0 (− 7.9 to – 4.1)	− 6.2 (− 7.3 to −5.0)	−3.5 (−5.9 to −1.1)	−2.9 (− 5.8 to −0.9) kg	− 3.2 (− 5.0 to – 1.4)
% Losing ≥5% weight n (%)	46 (57%)	26 (58%)	72 (57%)	7 (27%)	5 (19%)	12 (23%)

a Baseline observation carried forward weight loss mean change (95% CI)

### Breast Cancer risk categories

Uptake, retention and weight loss for the combined programmes across the four BC risk groups are reported in Table 3. Factors associated with uptake and retention are reported in Table 4.

**Table 3:** Uptake, retention and weight loss across breast cancer risk categories in the two studies

	Study-1 Women informed of their breast cancer risk prior to invite to the weight loss programme					Study-2 Women informed of their breast cancer risk part way through the weight loss programme				
10-year risk of breast cancer	Invited n	Uptake n (%)	Retention at 12 months n (%)	Weight loss kg^a^	% Losing ≥5% weight n (%)	Invited n	Uptake n (%)	Retention at 12 months n (%)	Weight loss kg ^a^	% Losing ≥5% weight n (%)
Low (< 2%)	560	28 (5)	15 (54)	−2.9 (−4.3 to −1.4)	12 (43)	541	26 (5)	7 (27)	−1.1 (−2.7 to + 0.5)	2 (8)
Average (< 5 to > 2%)	437	38 (9)	29 (76)	−5.9 (−7.8 to −3.9)	22 (58)	Not invited				
Moderately increased (≥5 to < 8%)	187	30 (16)	20 (67)	−7.0 (−9.9 to −4.1)	17 (57)	60	9 (15)	6 (60)	−5.3 (−13 to + 2.6)	3 (33)
High (≥8%)	172	30 (17)	22 (73)	−7.1 (−9.7 to −4.5)	21 (70)	137	17 (12)	11 (69)	−5.2 (−8.5 to −1.9)	7 (41)
Total/average	1356	126 (9)	86 (68)	- 6.2 (−7.3 to −5.0)	72 (57)	738	52 (7)	24 (46)	−3.2 (−5.0 to −1.4)	12 (23)

a Baseline observation carried forward weight loss mean change (95% CI)

**Table 4:** Factors associated with uptake and retention to the weight loss programmes using multivariable analysis

	Study-1: Women informed of their breast cancer risk prior to invite to the weight loss programme				Study-2: Women informed of their breast cancer risk part way through the weight loss programme			
	Uptake		Retention		Uptake		Retention	
	Odds ratio (95% CI)	*P* value	Odds ratio (95% CI)	*P* value	Odds ratio (95% CI)	*P* value	Odds ratio (95% CI)	*P* value
Breast Cancer Risk: High and moderately increased vs. low	1.99 (1.24 to 3.17)	**0.004**	2.98 (1.05 to 8.47)	**0.041**	3.58 (1.59 to 8.07)	**0.002**	3.88 (1.07 to 14.04)	**0.039**
Age per year	0.93 (0.90 to 0.96)	**0.000**	1.15 (1.03 to 1.28)	**0.011**	0.92 (0.80 to 1.07)	0.265	1.09 (0.92 to 1.30)	0.331
Index of multiple deprivation: Townsend quintile 1 vs. 3,4,5	2.1 (1.3to 3.3)	**0.009**	0.264 (0.076 to 0.920)	**0.050**	Not included in the model		Not included in the model	
Townsend quintile 2 vs. 3,4,5	1.8 (1.0 to 3.0)		0.234 (0.069 to 0.799)		Not included in the model		Not included in the model	
Time since receiving risk feedback (months)	1.00 (0.99 to 1.0)	0.387	Not included in the model		N/A		N/A	
Smoker vs. non-smoker	No smoking data on women who did not join the study		0.045 (0.006 to 0.353)	**0.003**	Not included in the model		Not included in the model	
BMI per unit increase	Not included in the model		0.82 (0.73 to 0.92)	**0.001**	Not included in the model		Not included in the model	

a Townsend quintile; 1 is the least deprived. N/A not applicable as risk feedback was received after women had decided to join Study 2. Figures in bold indicate significance (*P* value ≤ 0.05)

### Uptake

In Study-1 the odds of joining the study were significantly higher amongst high/moderately increased compared to low BC risk group low Townsend (least deprived) compared to high Townsend quintiles, and lower with increasing age. High and moderately increased risk women were 99% more likely to join than low risk women; odds ratio (95% confidence interval [CI] 1.99 (1.24 to 3.1), *P* = 0.004. Women in Townsend quintile 1 were 110% more likely; 2.1(1.3to 3.3) whilst those in quintile 2 were 80% more likely to join than women in groups 3, 4 and 5; 1.8 (1.0 to 3.0), *P* = 0.009. There was a 7% reduction in uptake per year of increasing age; 0.93 (0.90 to 0.96), *P* = 0.000) (Table 4). In Study-2 the odds of joining the study were significantly higher amongst high/moderately increased compared with low BC risk groups, although women had not yet been informed of these risks 3.58 (1.59 to 8.07), *P* = 0.002. However uptake was unrelated to age 0.92 (0.80 to 1.07),*P* = 0.265 (Table 4).

Cross study comparison showed a numerically higher uptake amongst high-risk women in Study-1 who had received risk feedback, compared to Study-2 who had not yet received this. Although though this did not reach significance (17 vs 12%; *P* = 0.221) (Table 3). Characteristics of women invited to Study 1 and Study 2 who joined and did not join the studies are reported in Additional file 1.

### Retention and weight loss

Retention at 12 months in Study-1 was greater in the high/moderately increased BC risk groups who were 198% more likely to remain on the programme compared to low risk women; odds ratio (95% CI) 2.98 (1.05 to 8.47), *P* = 0.041. There was a 15% greater retention per year of increasing age; 1.15 (1.03 to 1.28),*P* = 0.011. Retention was lower amongst heavier women, 18% lower retention for each unit increase in BMI; 0.82 (0.73 to 0.92),*P* = 0.001) and smokers who were 95% less likely to remain in the study compared to non-smokers; 0.045 (0.006 to 0.353), *P* = 0.003). The least deprived women in quintile 1 were 74% less likely to remain in the programme than women in quintiles 3, 4 and 5, 0.264 (0.076 to 0.920), whilst women in quintile 2 were 77% less likely to remain in the programme than women in quintiles 3, 4 and 5; 0.234 (0.069 to 0.799) *P* = 0.050, (Table 4). Retention was significantly lower amongst low-risk women in Study-2 where women had been informed of their low BC risk part way through the programme, compared to Study-1 (27% vs 54% *P* = 0.046) (Table 3).

Weight loss of ≥5% at 12-months was achieved by 63% high/moderately increased BC risk vs. 43% low risk in Study-1 (*P* = 0.083) and 39% high/moderately increased BC risk vs. 8% low risk in Study-2 (*P* = 0.008) (Table 3). No reported adverse events were considered to be related to trial participation.

## Discussion

BC risk status was an independent predictor of uptake, retention and weight loss with the weight loss programmes across both studies. The increased uptake amongst higher risk women was apparent in Study-2 before women had been informed of their risk, most likely due to their knowledge of BC history within the family. Many of the high-risk women had at least one first degree relative with BC (high-risk 83%, moderately increased risk 60% compared to 5.3 and 0% of women at average and low risk). Communicating personalised BC risk appeared to confer modest increases in uptake amongst high-risk individuals in Study-1 and led to disengagement from the programmes amongst those at low-risk in Study-2.

We have previously shown a positive association between BC risk and uptake of chemoprevention [27] and risk reducing mastectomy [28]. The novel finding that women at higher risk of BC have a better uptake, retention and weight loss success with a lifestyle weight loss programme across both studies is important. A recent systematic review reported that personalised cancer risk information did not increase smoking cessation, but had not been studied in the context of diet, PA and alcohol [9] as here.

Strengths of our studies include complete data on uptake and retention and key demographic factors. Limitations include that the analyses are exploratory, as the studies were not powered to test uptake and efficacy of the programmes. The relatively low overall uptake to both studies is typical of that seen with mailed invitations [29] which limits the generalisability of the findings. Uptake to our cohort was mainly White British, and from less deprived groups. This is consistent with lower uptakes to breast screening and cancer research amongst minority ethnic and low socioeconomic groups [5]. Future work needs to understand the best ways to engage these hard to reach populations with screening and prevention. A final limitation is that body weight was assessed in clinic by researchers who were aware of their allocation to the breast cancer or multiple disease prevention programme but were blinded to their level of breast cancer risk.

The increased engagement amongst high and moderately increased risk women can be utilised to target women for whom programmes are likely to provide greater risk reductions [30] and be more cost effective. However, the apparent disengagement of low risk women is a concern given the potential widespread disease prevention and well-being benefits of a healthy lifestyle across all BC risk categories [31]. This would need to be addressed if BC risk feedback is provided to screening populations.

## Conclusion

Women at increased risk of BC in the NHSBSP are significantly more likely to engage and be successful with a weight loss programme at 12 months than women at low risk. Thus it is possible to target high risk women in a Breast Screening Programme who have the greatest potential benefit fom risk reduction strategies. Future research will test the cost effectiveness of the weight loss programmes amongst the 15% of screening attendees identified at high/moderately-risk of BC.

## Supplementary information

**Additional file 1.** Characteristics of women who joined and did not join Study 1 and Study 2.

## Data Availability

The trial protocol and all datasets used and analysed during the current study are available from the corresponding author on reasonable request**.**

## Abbreviations

BC: Breast cancer
BMI: Body mass index
BOCF: Baseline observation carried forward
CVD: Cardiovascular disease
HbA1c: Glycated haemoglobin
NHSBSP: NHS Breast Screening Programme
PA: Physical activity
PROCAS: Predicting of Cancer at Screening
T2D: Type 2 diabetes
