# Supplementary material for: Breast cancer risk status influences uptake, retention and efficacy of a weight loss programme amongst breast cancer screening attendees: two randomised controlled feasibility trials
Source: BMC Cancer. 2019 Dec 4;19:1089. doi: 10.1186/s12885-019-6279-8 (PMC6892016; doi:10.1186/s12885-019-6279-8)
Supplement: Supplementary file 1 — Additional file 1. Characteristics of women who joined and did not join Study 1 and Study 2. [file 12885_2019_6279_MOESM1_ESM.docx]

Additional file 1

Characteristics of women who joined and did not join Study 1 and Study 2

|  | Study 1 | | Study 2 | |
| --- | --- | --- | --- | --- |
|  | Women recruited to the study  (n =126) | Women not recruited to the study  (n =1230) | Women recruited to the study  (n =52) | Women not recruited to the study  (n =680) |
| Age (years)^a^ | 59.0 (5.1) | 61.7 (5.7) | 53.3 (4.3) | 54.0 (4.3) |
| BMI (kg/m^2^)^a^ | 31.4 (4.5) | 30.9 (5.3) | 31.1 (4.8) | 30.1 (5.7) |
| Townsend quintile (%):  1 (least deprived)  2  3  4  5 (most deprived) | 32  41  20  5.5  1.5 | 21  30  31  12  6 | 48  26  17  8  4 | 32  30  26  12  0.4 |
| Ethnicity (%):  White British  Asian  Afro-Caribbean | 97.0  1.5  1.5 | 91.8  3.0  5.2 | 98.0  1  1 | 97.8  1.9  1.3 |
| Number of first degree relatives with breast cancer (%)  0  1  >or=2 | 68.3  28.6  3.2 | 79.2  17.8  3.0 | 76.9  23.1  0.0 | 80.8  17.3  1.9 |
| Time since receiving risk feedback (months)^b^ | 12.0 (21.6) | 8.4(12.9) | N/A | N/A |

a Mean (SD) b Median (interquartile range) N/A not applicable
